# Low lung function in Bipolar Disorder and Schizophrenia: a hidden risk

**DOI:** 10.3389/fphys.2024.1335798

**Published:** 2024-04-25

**Authors:** Cristina Ruiz-Rull, María José Jaén-Moreno, Gloria Isabel del Pozo, Cristina Gómez, Francisco Javier Montiel, Montserrat Alcántara, Laura Carrión, Geli Marie Chauca, Nuria Feu, Ipek Guler, Fernando Rico-Villademoros, Cristina Camacho-Rodríguez, Luis Gutierrez-Rojas, David Mannino, Fernando Sarramea

**Affiliations:** ^1^ Instituto Maimónides de Investigación Biomédica de Córdoba (IMIBIC), Córdoba, Spain; ^2^ Centro de Salud Cruz de Caravaca, Almería, Spain; ^3^ Departamento de Ciencias Morfológicas y Sociosanitarias, Universidad de Córdoba, Córdoba, Spain; ^4^ Unidad de Gestión Clínica de Salud Mental, Hospital Universitario Reina Sofía, Córdoba, Spain; ^5^ Unidad de Gestión Clínica de Salud Mental, Complejo Hospitalario de Jaén, Jaen, Spain; ^6^ Unidad de Gestión Clínica de Salud Mental, Hospital Infanta Margarita, Cabra, Spain; ^7^ Unidad de Gestión Clínica de Neumología, Hospital Universitario Reina Sofía, Córdoba, Spain; ^8^ Instituto Maimónides de Investigación Biomédica de Córdoba (IMIBIC), Área de Gestión de la Investigación, Córdoba, Spain; ^9^ Instituto de Neurociencias, Universidad de Granada, Granada, Spain; ^10^ Departamento de Psiquiatria, Universidad de Granada, Granada, Spain; ^11^ University of Kentucky, Chief Medical Officer, COPD Foundation, Lexington, KY, United States; ^12^ Centro de Investigación Biomédica en Red de Salud Mental (CIBERSAM), Oviedo, Spain

**Keywords:** serious mental illness, schizophrenia, bipolar disorder, spirometry, obstructive, restrictive, preserved ratio impaired spirometry

## Abstract

**Introduction:** People with serious mental illness (SMI), such as schizophrenia and bipolar disorder, have a higher risk of premature morbidity and mortality. In the general population, impaired lung function is associated with increased morbidity and mortality. We compared lung function between people with and without serious mental illnesses using a cross-sectional study in 9 community mental health units.

**Methods:** Subjects aged 40–70 years with a diagnosis of schizophrenia or bipolar disorder were recruited consecutively. The controls had no psychiatric diagnosis and were not receiving any psychotropics. Spirometry was performed by a trained nurse. We used the 2021 American Thoracic Society/European Respiratory Society standards for the interpretation of the spirometry results.

**Results:** We studied 287 subjects. People with SMI (*n* = 169) had lower spirometry values than those without a psychiatric diagnosis (*n* = 118). An abnormal spirometry pattern (36.1% vs 16.9%, *p* < 0.001), possible restriction or non-specific (Preserved Ratio Impaired Spirometry [PRISm]) pattern (17.8% vs 7.6%, *p* = 0.014), and pattern of airflow obstruction or possible mixed disorder (18.3% vs 9.3%, *p* = 0.033) were more frequent in people with SMI. Multivariate analyses showed that the PRISm pattern was associated with abdominal circumference (odds ratio [OR] 1.05, 95%CI 1.03–1.08) and that the pattern of airflow obstruction or possible mixed disorder was associated with smoking behavior (OR 5.15, 95%CI 2.06–15.7).

**Conclusion:** People with SMI have impaired lung function, with up to one-third of them showing an abnormal spirometry pattern. This suggests that regular monitoring of lung function and addressing modifiable risk factors, such as tobacco use and obesity, in this population is of paramount importance.

## 1 Introduction

People with serious mental illness (SMI), such as schizophrenia and bipolar disorder, have a higher risk of premature morbidity and mortality compared to the general population. It has been observed that their life expectancy may be reduced by 12–15 years in people with schizophrenia (Crump et al., 2013a) and approximately 9 years in people with bipolar disorder (Crump et al., 2013b). This is a major public health issue that requires intervention (Liu et al., 2017; Fiorillo and Sartorius, 2021) and improved knowledge of the risk factors. In relative terms, unnatural causes of death, such as suicide, are associated with the highest excess mortality in people with SMI (Saha et al., 2007; Hayes et al., 2015). Among natural causes, chronic obstructive pulmonary disease (COPD) is one of the highest causes of excess mortality in people with schizophrenia or bipolar disorder, even higher than that of cardiovascular diseases (Saha et al., 2007; Hayes et al., 2015; Olfson et al., 2015; Correll et al., 2022). In the general population, both COPD and spirometrically determined impaired lung function are associated with increased morbidity and mortality (Sin et al., 2005; Guerra et al., 2010; Agusti et al., 2017; Honda et al., 2017; Cuttica et al., 2018; Collaro et al., 2021; Guo et al., 2021; Shah et al., 2021; Kaaks et al., 2022; Ramalho et al., 2022; Sarycheva et al., 2022; Dharmage et al., 2023).

Spirometry is a noninvasive, reproducible and readily available test for evaluating lung function (Global Initiative for Chronic Obstructive Lung Disease, 2022) and has been shown to be a key tool in diagnosing lung disease and monitoring patients (Lamb et al., 2022). To date, four studies have evaluated lung function using spirometry in patients with SMI (Filik et al., 2006; Vancampfort et al., 2014a; Vancampfort et al., 2014b; Partti et al., 2015; Jaen-Moreno et al., 2021). Filik et al. (2006) and Vancampfort et al. (2014a); Vancampfort et al. (2014b) reported that people with schizophrenia had lower lung function as evaluated by spirometry, reporting a lower forced expiratory volume in 1 s (FEV1) and lower forced vital capacity (FVC); however, these studies did not report the frequency of obstructive and restrictive patterns. Using baseline data from a randomized controlled trial in active smokers, Jaen-Moreno et al. (2021) secondarily reported that 27 (23.9%) of 113 patients with SMI met the Global Initiative for Chronic Obstructive Lung Disease (GOLD) criteria for COPD, and among 86 patients who did not meet COPD criteria, 23 (26.7%) showed an FEV1 less than 80% of the predicted value, which is consistent with a restrictive pattern. Finally, Partti et al. (2015), in a population-based study, showed that people with schizophrenia and other nonaffective psychiatric disorders (e.g., schizophreniform disorder) had impaired lung function and a higher likelihood of a restrictive pattern than people with no psychiatric disorder, and people with schizophrenia also had a higher likelihood of an obstructive pattern. Data on lung function in people with bipolar disorder are lacking.

Our study aimed to evaluate lung function and the prevalence of abnormal spirometry patterns, and explore the factors associated with them in people with SMI (schizophrenia or bipolar disorder) in a community-based mental healthcare sample and compare it to that of people without a psychiatric diagnosis.

## 2 Materials and methods

This was an observational cross-sectional study conducted in 9 community mental health units in Andalucía (southern Spain). A control group was used to increase the internal validity of the study and support the causal inference. The study was approved by the Ethics Committee of Reina Sofía Hospital (Córdoba, Spain), and written informed consent was obtained from all subjects.

### 2.1 Selection criteria

We recruited consecutive subjects attending scheduled follow-up visits at their mental health outpatient service. The subjects had to be 40–70 years of age. We included subjects aged 40 years or older because the harmful effects of tobacco occur from that age (Górecka et al., 2003) and excluded subjects over 70 years old because, due to shortened life expectancy, it is difficult to find people with SMI who are attending a psychiatric outpatient clinic. In the clinical sample (i.e., patients with a psychiatric diagnosis), additional inclusion criteria were a DSM-IV diagnosis of schizophrenia or bipolar disorder and clinical stability, which was defined as a score lower than 14 points on the Hamilton Depression Rating Scale (HDRS) and a score lower than six points on the Young Mania Rating Scale (YMRS) in people with bipolar disorder or a score lower than 40 points on the Positive and Negative Syndrome Scale (PANSS) in people with schizophrenia. Subjects without a psychiatric diagnosis (controls) were recruited from those accompanying the person with a psychiatric diagnosis and from those visiting the primary care center for administrative purposes; they were excluded if they had any current or past diagnosis of a psychiatric disorder or were receiving any psychotropic.

In both samples, with or without psychiatric diagnosis, subjects were excluded if they exhibited a clinical condition that made it advisable not to perform spirometry (recent pneumothorax, recent thoracic or abdominal surgery, aortic aneurysm, unstable angulation, retinal detachment, facial hemiparesis or oral/dental problems), had an intellectual disability or a clinical status that, in the investigator’s judgment, made the subject unable to properly understand the instructions for forced spirometry or if they had history of respiratory diseases.

### 2.2 Assessments

We recorded sociodemographic data, anthropometric data including abdominal circumference, and vital signs (i.e., heart rate and blood pressure). We included abdominal circumference because it is considered that body mass index alone is not sufficient to properly assess the cardiometabolic risk associated with increased adiposity and, therefore, it is recommended as a routine vital sign measurement (Ross et al., 2020). In addition, some studies suggest that abdominal circumference is inversely associated with FEV1 (Wehrmeister et al., 2012). In people with a psychiatric diagnosis, we administered the HDRS, YMRS and PANSS, whereas in controls, the lack of psychiatric diagnosis and lack of history of psychiatric treatment/follow-up were confirmed. The following medical comorbidities were searched from the medical charts and recorded: hypertension, diabetes, dyslipidemia, peripheral vascular disease, heart disease and oncological disease. The evaluation of smoking habit included the history of consumption, current consumption confirmed by CO-oximetry, and dependence on nicotine as evaluated with the Fagerström Test for Nicotine Dependence (Becoña and Vázquez, 1998) (FTND), a standard instrument for assessing the intensity of physical addiction to nicotine which categorizes dependence as mild (0–3), moderate (Saha et al., 2007; Hayes et al., 2015; Liu et al., 2017; Correll et al., 2022) and severe (Olfson et al., 2015; Agusti et al., 2017; Collaro et al., 2021). Physical activity was estimated using the Spanish validated version of the International Physical Activity Questionnaire (IPAQ) (Roman et al., 2006), which allows the calculation of energy expenditure as metabolic equivalents of task (METs) and the total expenditure as METs-minute/week as well as a sedentary index as the total time in minutes seated in a week.

Spirometry was performed at each participant site using a DATOSPIR Touch Easy D+ spirometer (Sibelmed^®^, Barcelona, Spain). To validate the equipment used and check its characteristics, the test and criteria for quality control was used for acceptability and repeatability of the results; the standardization criteria set down by the American Thoracic Society and the European Respiratory Society (ATS/ERS) (Miller et al., 2005). They were performed by a nurse trained by the pulmonology service of the Reina Sofía Hospital (Córdoba, Spain) on calibration, preparing patients, performing maneuvers, bronchodilatation and repetition of spirometry. A maximum of eight efforts could be made to achieve a minimum of three acceptable blows. The system automatically selected the two best curves with repeatability criteria, that is, the two highest FVC values and the two highest FEV1 values, with a difference of less than 0.15 L. The reversibility test required the repetition of three acceptable efforts 15 min after the inhalation of a bronchodilator (salbutamol 400 µg). Curves and volumes were centrally assessed by a single blinded researcher, the head of the functional test unit of the pulmonology service of the Reina Sofía Hospital (Córdoba, Spain), and the highest readings of FVC and FEV1 were recorded and used in the analysis. Following the 2021 American Thoracic Society (ATS) and European Respiratory Society (ERS) standards (Stanojevic et al., 2022), z-scores were calculated using GLI reference equations, and FEV1/FVC ratio <fifth percentile (z-score < -1.645) and FVC >fifth percentile (z-score > -1.645) were considered indicative of airflow obstruction; FEV1/FVC ratio <fifth percentile (z-score < -1.645) and FVC <fifth percentile (z-score < -1.645) were considered indicative of a possible mixed disorder; and a FEV1/FVC ratio >fifth percentile (z-score > -1.645) and a FVC <fifth percentile (z-score < -1.645) were indicative of a possible restriction or non-specific pattern (Preserved Ratio Impaired Spirometry [PRISm]). The severity of lung function (for all measures using the z-score) was considered mild (−1.65 to −2.5), moderate (−2.51 to −4.0) or severe (<-4.1). According to the 2021 ERS/ATS standards, the response to bronchodilation was considered positive when the difference between pre-bronchodilation and post-bronchodilation was greater than 10% of predicted value in FEV1 or FVC. In addition, predicted values were calculated as a percentage of the reference values from the Spanish Society of Pneumology and Thoracic Surgery (*Sociedad Española de Neumología y Cirugía Torácica* [SEPAR]), enabling comparison with the existing literature for this particular population group.

### 2.3 Statistical analysis

Continuous variables were expressed as the mean and standard deviation or the median and range and compared using the Wilcoxon test. Cohen’s d effect size was calculated as the quotient between the difference in means and pooled standard deviation of the two groups. To interpret the relative magnitude of the difference, we considered effect sizes of 0.20 as small, 0.50 as moderate and 0.80 or greater as large. Categorical variables were expressed as absolute and relative frequencies and compared using the chi-squared test. Due to the sample size of the subgroups and consistent with the protocol, all comparisons were limited to those between the overall group of people with SMI vs the control group; however, descriptive data are also presented for the subgroups of people with schizophrenia and people with bipolar disorder.

To evaluate the potential factors associated with spirometry patterns, multivariate logistic regression models were independently fitted for each ATS/ERS spirometry pattern categorized as airflow obstruction or possible mixed disorder (hereafter referred to obstructive component pattern) and possible restriction or non-specific pattern (PRISm). Additionally, we considered an abnormal pattern that combined the two previous patterns. Variable selection was performed using the Akaike Information Criterion (AIC) with backward stepwise elimination in a multivariate model setting using the variables suggested in the literature as risk factors.

The results are expressed as odds ratios (ORs), 95% confidence intervals (CIs) and *p* values.

All statistical analyses were performed using R software, version 4.0.3.

## 3 Results

### 3.1 Patient disposition and characteristics

From July 2018 to February 2020, we recruited 287 participants: 169 with SMI (111 with schizophrenia and 58 with bipolar disorder) and 118 controls. Compared to controls, those with SMI were younger, had a greater waist circumference, body mass index, and sedentary index, had a higher proportion of smokers, were heavier smokers and showed greater nicotine dependence, as shown by the FTND score (Table 1). People with SMI also had a higher frequency of diabetes (14.4% vs 6.1%, *p* = 0.049) and a lower frequency of hypertension (10.7% vs 25.4%, *p* = 0.002) (Table 1).

**TABLE 1:** Demographic and clinical characteristics.

	Serious mental illness			Controls	
Variable	Total	Schizophrenia	Bipolar disorder		*p*-value^a^
	N = 169	N = 111	N = 58	N = 118	
Age, mean (SD)	50.5 (6.60)	49.3 (6.26)	52.8 (6.70)	52.3 (7.09)	0.044
Gender, n (%)					0.138
Female	60 (49.8)	31 (27.9)	38 (65.5)	59 (50.4)	
Male	100 (59.2)	80 (72.1)	20 (34.5)	58 (49.6)	
Abdominal circumference, mean (SD)	107 (14.1)	107 (14.4)	106 (13.5)	101 (16.4)	0.002
Body mass index, mean (SD)	30.6 (5.64)	30.1 (5.61)	31.6 (5.63)	29.2 (5.68)	0.040
Education level, n (%)					0.007
None	13 (7.7)	9 (8.1)	4 (7)	3 (2.6)	
Primary	83 (49.4)	57 (51.4)	26 (45.6)	43 (37.4)	
Secondary	55 (32.7)	33 (29.7)	22 (38.6)	44 (38.3)	
University	17 (10.1)	12 (10.8)	5 (8.8)	25 (21.7)	
Net monthly income, mean (SD)	680.1 (434.8)	616.3 (312.2)	845.6 (631.2)	1366.5 (463)	<0.001
Population size, n (%)					<0.001
<10.000	76 (45.2)	49 (44.1)	27 (47.4)	73 (64)	
10.000–50.000	44 (26.2)	26 (23.4)	18 (31.6)	6 (5.3)	
50.000–250.000	19 (11.3)	15 (13.5)	4 (7)	1 (0.9)	
>250.000	29 (17.3)	21 (18.9)	8 (14)	34 (29.8)	
Physical activity (IPAQ), median (range)					
Activity (METS)	693 (198; 1386)	693 (198; 1386)	693 (297; 1378)	693 (173; 1386)	0.592
Sedentary index	1260 (840; 2100)	1260 (840; 2100)	1680 (840; 2100)	840 (630–1260)	<0.001
Arterial hypertension (Yes), n (%)	18 (10.7)	11 (9.91)	7 (12.3)	29 (25.4)	0.002
Diabetes mellitus (Yes), n (%)	24 (14.4)	13 (11.8)	11 (19.3)	7 (6.14)	0.049
Dyslipidemia (Yes), n (%)	32 (19.0)	19 (17.1)	13 (22.8)	21 (18.4)	1.000
Peripheral vascular disease (Yes), n (%)	6 (3.6)	4 (3.6)	2 (3.6)	2 (1.7)	0.481
Heart disease (Yes), n (%)	2 (1.2)	1 (0.9)	1 (1.8)	4 (3.5)	0.227
Oncological disease (Yes), n (%)	2 (1.2)	0 (0.0)	2 (3.5)	3 (2.6)	0.397
Smoking (Yes), n (%)	117 (69.2)	86 (77.5)	31 (53.4)	49 (41.5)	<0.001
Tobacco variables^b^					
Cigarettes per day, mean (SD)	23.4 (10.2)	23.4 (10.6)	23.1 (9.37)	19.5 (8.76)	0.010
Cigarettes pack/year, mean (SD)	36.6 (17.8)	36.1 (18.1)	38.0 (17.2)	30.6 (17.2)	0.022
Years of tobacco consumption, mean (SD)	31.6 (7.96)	31.1 (7.79)	33.1 (8.36)	30.6 (6.48)	0.384
Expired carbon monoxide (CO), mean (SD)	13.7 (10.0)	15.1 (9.74)	11.0 (10.1)	5.9 (6.85)	<0.001
FTND mean (SD)	5.97 (1.99)	6.07 (1.92)	5.68 (2.17)	4.04 (1.34)	<0.001

FTND, Fagerström Test for Nicotine Dependence; IPQ, international physical activity; MET, metabolic equivalent of task; SD, standard deviation.

^a^ For the comparison of the overall severe mental illness group vs the general population.

^b^ Evaluated only among smokers.

### 3.2 Spirometry results

People with SMI had significantly lower FEV1 z-score, FVC z-score, and FEV1/FVC ratio z-score than controls; effect sizes as evaluated with Cohen’s d were moderate for the differences in the FEV1 and FVC z-scores, and small for the difference in FEV1/FVC z-score (Table 2). When evaluated with FEV1 z-score, 24 (14.2%) subjects with SMI showed a moderate lung function impairment and 1 (0.6%) had a severe impairment, while there were no cases of severe impairment and there was one case of moderate lung function impairment among controls (Table 2). Except for the effect sizes, % predicted values showed similar results (Table 2; Supplementary Table S1).

**TABLE 2:** Spirometry results.

Variable	Serious mental illness				Between group differences		
					(SMI total vs Controls)		
	Total (N = 169)	Schizophrenia (N = 111)	Bipolar disorder (N = 58)	Controls	Difference	Cohen’s d	*p*-value
				(N = 118)	(95% CI)	(95% CI)	
**FEV1, liters: mean (SD)**	2.70 (0.76)	2.85 (0.76)	2.44 (0.68)	2.95 (0.69)	0.25	0.34	0.008
					(0.08, 0.42)	(0.11, 0.58)	
**FEV1, % predicted: mean (SD)**	85.5 (17.5)	84.6 (19.1)	87.2 (14.2)	101 (16.7)	15.5	0.90	<0.001
					(11.44, 19.56)	(0.67, 1.14)	
**FEV1 z-score: mean (SD)**	−1.26 (1.15)	−1.27 (1.22)	−1.25 (1.01)	−0.51 (0.95)	0.75	0.7	<0.001
					(0.51, 0.99)	(0.46, 0.95)	
**FVC, liters: mean (SD)**	3.60 (0.97)	3.80 (0.96)	3.22 (0.86)	3.84 (0.91)	0.24	0.25	0.034
					(0.02, 0.46)	(0.02, 0.49)	
**FVC, % predicted: mean (SD)**	88.7 (15.7)	88.2 (16.5)	89.8 (14.1)	103 (16.4)	14.3	0.89	<0.001
					(10.52, 18.08)	(0.66, 1.13)	
**FVC, z-score: mean (SD)**	−0.94 (1.09)	−0.92 (1.16)	−0.97 (0.94)	−0.30 (0.95)	0.64	0.62	<0.001
					(0.40, 0.88)	(0.38, 0.86)	
**FEV1/FVC, z-score: mean (SD)**	−0.66 (1.08)	−0.72 (1.06)	−0.56 (1.12)	−0.34 (1.02)	0.32	0.31	0.011
					(0.08, 0.57)	(0.07, 0.54)	
**Severity of lung function impairment (based on FEV1 z-scores), n (%)**							
**Normal**	104 (61.5)	67 (60.4)	37 (63.8)	108 (91.5)			
**Mild**	40 (23.7)	26 (23.4)	14 (24.1)	9 (7.6)			
**Moderate**	24 (14.2)	17 (15.3)	7 (12.1)	1 (0.8)			
**Severe**	1 (0.6)	1 (0.9)	0 (0.0)	0 (0.0)			

BD, bronchodilatation; FEV1, forced expiratory volume in 1 s; FVC, forced vital capacity; SD, standard deviation; SMI, serious mental illness.

**FIGURE 1:**
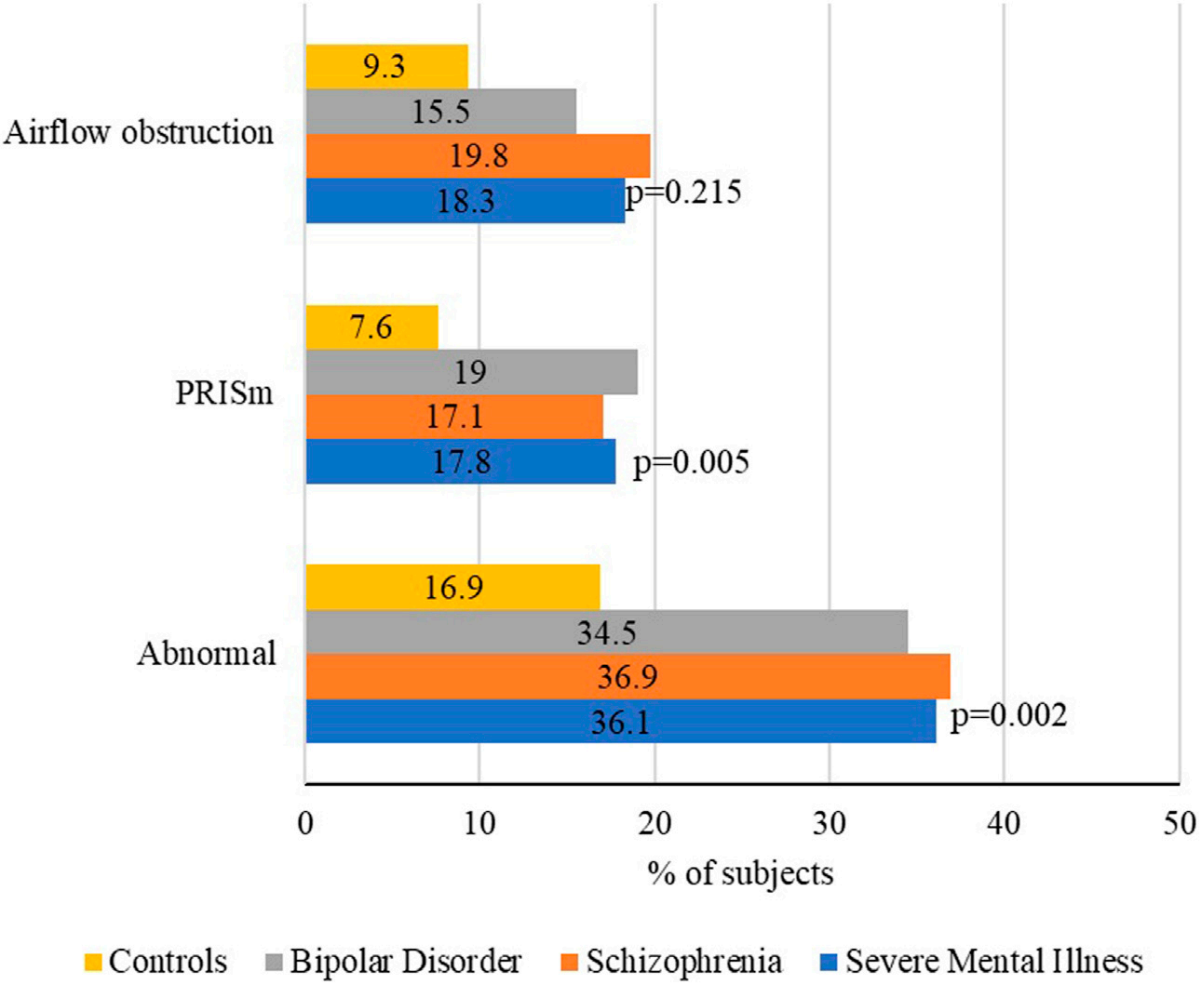
Spirometric patterns. *p*-values refer to the comparison between serious mental illness and the general population.

Overall, 61 (36.1%) of the people with SMI had an abnormal spirometry pattern compared to 20 (16.9%) of controls (*p* < 0.001). The PRISm pattern was significantly more frequent in people with SMI than controls (17.8% vs 7.6%, *p* = 0.014), and the proportion of subjects showing an obstructive component pattern was also significantly higher among people with SMI (18.3% vs 9.3%, *p* = 0.033) (Figure 1). Of the 31 subjects with SMI and obstructive component pattern, 1 (3.2%) responded to bronchodilators; 4 (36.4%) of 11 controls with an obstructive component pattern responded to bronchodilators Spirometry patterns according to the GOLD criteria are shown in Supplementary Figure S1.

### 3.3 Multivariate analyses

The factors associated with a greater likelihood of presenting an abnormal spirometry pattern were having a SMI (odds ratio [OR] 2.29, 95% CI 1.26 to 4.28; *p* = 0.008) and smoking (OR 2.19, 95% CI 1.20–4.07) (Table 3). Smoking was significantly associated with an obstructive component pattern (OR 5.15, 95% CI 2.06–15.7) and the abdominal perimeter was significantly associated with a PRISm pattern (OR 1.05, 95% CI 1.03–1.08) (Table 3).

**TABLE 3:** Multivariate analyses.

Abnormal spirometric pattern			
Characteristic	OR	95% CI	*p*-value
**Age**	0.99	0.95 to 1.03	0.6
**Gender**			0.3
Female	-	-	
Male	1.37	0.79 to 2.40	
**Group**			0.008
Controls	-	-	
SMI	2.29	1.26 to 4.28	
**Smoking**			0.011
No	-	-	
Yes	2.19	1.20 to 4.07	

Air Flow obstruction pattern or Possible mixed disorder			
(obstruction component pattern)			
**Age**	0.97	0.92 to 1.02	0.3
**Gender**			0.2
Female	-	-	
Male	1.55	0.75 to 3.31	
**Group**			0.3
Controls	-	-	
SMI	1.48	0.68 to 3.45	
**Smoking**			0.001
No	-	-	
Yes	5.15	2.06 to 15.7	

PRISm			
(possible restriction or non-specific pattern)			
**Age**	1.00	0.95 to 1.06	0.9
**Gender**			0.6
Female	-	-	
Male	0.82	0.39 to 1.74	
**Group**			0.07
Controls	-	-	
SMI	2.19	0.97 to 5.34	
**Smoking**			0.9
No	-	-	
Yes	0.95	0.44 to 2.09	
**Abdominal perimeter**	1.05	1.03 to 1.08	<0.001

PRISm, Preserved Ratio Impaired Spirometry; SMI, serious mental illness.

## 4 Discussion

We found that people with SMI showed a significant reduction in all spirometric values and had an increased frequency of an abnormal spirometry pattern, with significantly higher frequency of obstructive component and PRISm patterns than those without a psychiatric diagnosis. Among modifiable risk factors, while smoking was the main risk factor for an obstructive component pattern, abdominal obesity was the main factor associated with the presence of the PRISm pattern.

People with SMI showed a significant impairment of lung function as evaluated by FEV1 and FVC z-scores, and substantial proportion of subjects (15%) showed at least moderate impairment compared to almost no cases (1%) among controls. None of the previously published studies on lung function in people with SMI used the new ATS/ERS standards for the analyses of spirometry; therefore, in some instances to put our results into perspective we will use % predicted values and the GOLD criteria for obstructive and restrictive patterns. When using classical spirometry parameters, our results are consistent with those reported by Partti et al. and Vancampfort et al. (2014a) in patients with schizophrenia. To the best of our knowledge, there are no previous reports on lung function in people with bipolar disorders.

We found that an obstructive component pattern was more frequent among subjects with SMI than among those without a psychiatric disorder. Our results are consistent with those reported by Partti et al. (2015) for the subgroup of patients with schizophrenia using the GOLD criteria. Furthermore, they are also consistent with the high rates of tobacco consumption in this population who show earlier age of smoking, and greater intensity and frequency of smoking (Dickerson et al., 2018).

The prevalence of the PRISm pattern in subjects with SMI was higher than that in controls. These results were consistent with those reported in a recent cross-sectional study conducted in people who were diagnosed with first-episode psychosis 10-year earlier, which found that the prevalence of PRISm was higher among people with psychosis compared to healthy controls (10.4% vs 1.4%) (Viejo Casas et al., 2024). The absolute difference between people with SMI and controls in our study for a PRISm pattern (17.8% vs 7.6%) was similar to that reported in this latter study; the higher frequency of PRISm in our study could be partially explained by the fact that the subjects included in our study were older (mean age 50.5 vs 41.7 years) and our definition of PRISm was based on z-scores. In our multivariate analysis, abdominal circumference was significantly associated with a higher likelihood of presenting a PRISm pattern. Our results are supported by the strong association found between obesity with the presence of PRISm in the general population (Wan et al., 2021; Higbee et al., 2022). It is also possible that antipsychotic treatment plays a role on the impairment of lung function. Although evidence is weak, some studies suggest that antipsychotics may be associated with an increased risk of acute respiratory failure (Wilson and Ridley, 2007; Wang et al., 2017). More importantly, several antipsychotics are associated with an increased risk of weight gain and other metabolic disorders (Pillinger et al., 2020; Bernardo et al., 2021); thus, they may contribute to the increased risk of PRISm in this population.

Several longitudinal studies have shown an association between reduced lung volume or abnormal spirometry patterns and increased morbidity and mortality. Reduced FEV1 has been associated with an increased risk of occurrence of respiratory disease (Agusti et al., 2017; Shah et al., 2021) and metabolic abnormalities, including diabetes (Agusti et al., 2017; Shah et al., 2021), heart failure (Ramalho et al., 2022) and cardiovascular events (Agusti et al., 2017; Cuttica et al., 2018), and an increased risk of cardiovascular (Sin et al., 2005) and all-cause mortality (Agusti et al., 2017; Sarycheva et al., 2022). COPD or a reduced FEV1/FVC ratio has been associated with an increased risk of lung cancer (Kaaks et al., 2022) and all-cause mortality (Collaro et al., 2021; Guo et al., 2021; Kaaks et al., 2022). Importantly, some of these studies have also shown the presence of a gradient in the relationship between reduced FEV1 (Sarycheva et al., 2022) or COPD (Guo et al., 2021; Kaaks et al., 2022) and all-cause mortality. A restrictive pattern has been associated with an increased risk of cardiovascular events (Guerra et al., 2010; Dharmage et al., 2023), hypertension (Dharmage et al., 2023), diabetes (Guerra et al., 2010; Dharmage et al., 2023) and cardiovascular and all-cause mortality (Guerra et al., 2010; Honda et al., 2017; Guo et al., 2021). PRISm has been also associated with an increased risk of all-cause mortality (Yang et al., 2023).

Our study has several limitations that should be considered. First, we performed a cross-sectional analysis, and thus, we cannot infer causality. Using data from a national health survey in the United States, Goodwin et al. (Goodwin et al., 2007) found an association between the presence of an obstructive and restrictive spirometry pattern and an impairment of mental health as evaluated by the General Wellbeing scale; after controlling for differences in demographic factors, there was an association between a spirometry restrictive pattern and lower scores in general health, vitality and self-control and higher scores for depression. Although we cannot rule out a bidirectional relationship between SMI and lung function impairment, there is evidence from longitudinal studies supporting the association of SMI with the occurrence of COPD and other respiratory diseases (Jaén-Moreno et al., 2023). Second, we used convenience sampling for the selection of the control group, based on the people accompanying the person with a psychiatric diagnosis or those visiting the primary care center for administrative purposes. Having a family member or a friend who is a smoker is a risk factor for being a smoker (Karadogan et al., 2018), which could explain why the prevalence of smokers in our control group was higher than in the general population (19.7% in 2019) (Eurostat, 2023). This could have biased the results against a potential association between SMI and lung function impairment. Third, in addition to a sedentary lifestyle, several medical comorbidities, such as hypertension, dyslipidemia and diabetes, have been associated with a spirometric restrictive pattern both in the general population (Backman et al., 2016; Sperandio et al., 2016) and in subjects with SMI (Partti et al., 2015). These medical comorbidities in our study were elicited through clinical charts and may be underdiagnosed in patients with SMI (Teixeira et al., 2022), thus limiting our multivariate analyses. Importantly, all these studies showing the association between medical comorbidities and impaired lung function were cross-sectional; therefore, we cannot be sure of the direction of the relationship. In a recent longitudinal study, the Tasmanian Longitudinal Health Study (Australia), subjects with a lifetime spirometry restrictive pattern were at increased risk of medical multimorbidity by middle age, including angina/myocardial infarction, hypertension, diabetes and sleep apnea (Dharmage et al., 2023). It is possible that lung function impairment and all medical comorbidities in patients with SMI share a common pathophysiological pathway (Teixeira et al., 2022). In the Tasmanian Longitudinal Health Study, subjects with a spirometry restrictive pattern showed the highest levels of C-reactive protein in middle age (Dharmage et al., 2023). Therefore, it is possible SMI will be associated with lung function impairment through intermediate variables such a smoking and abdominal obesity, direct mechanism such as a hyperinflammatory state, or a complex interplay of several factors. Fourth, information on exposure to inhaled toxicants in the workplace or related to traffic is unavailable. Finally, the age selection criterion could impact the generalizability of the results.

In our view, our study has some strengths. In contrast to Partti et al. (2015) and similar to Vancampfort et al. (2014a) we used a clinical sample and therefore were more representative of clinical practice. In addition, we used the new ATS/ERS standards for the analysis, and, although it was not the primary objective of our study, to our knowledge, we provide for the first time data on lung function in people with bipolar disorder.

Overall, consistent with what has been reported previously in a population-based study (Partti et al., 2015), our results show that people with SMI seen in mental health outpatient services have a significant and clinically relevant reduction in FEV1 and FVC, and suggest that up to one-third of people with SMI have an abnormal spirometry pattern, which in the case of the obstructive component pattern is associated with smoking and in the PRISm pattern with abdominal obesity. Although it has been infrequently studied in patients with SMI, the potentially severe consequences of an abnormal spirometry pattern suggest that patients with SMI should undergo regular monitoring of lung function to detect these alterations. This policy would be consistent with GOLD’s proposal for active COPD case finding (Global Initiative for Chronic Obstructive Lung Disease, 2022), bearing in mind that having an SMI—in addition to being a smoker—is a risk factor for lung function impairment. In a recent review of physical health guidelines for people with SMI, none of the 15 guidelines reviewed included recommendations for evaluating lung function (Friend et al., 2020). In addition to monitoring lung function, addressing modifiable risk factors for lung function impairment, such as tobacco use and obesity, which are also risk factors for other important and frequent comorbidities in people with SMI, is of paramount importance.

## Data Availability

The raw data supporting the conclusion of this article will be made available by the authors, without undue reservation.

## Abbreviations

AIC, Akaike Information Criterion; CI, confidence intervals; COPD, chronic obstructive pulmonary disease; FEV1, forced expiratory volume in 1 s; FVC, forced vital capacity; FTND, Fagerström Test for Nicotine Dependence; GOLD, Global Initiative for Chronic Obstructive Lung Disease; HDRS, Hamilton Depression Rating Scale; IPAQ, International Physical Activity Questionnaire; METs, metabolic equivalents of task; OR, odds ratio; PANSS, Positive and Negative Syndrome Scale; SMI, serious mental illness; YMRS, Young Mania Rating Scale.
