# Supplementary material for: Low lung function in Bipolar Disorder and Schizophrenia: a hidden risk
Source: Front Physiol. 2024 Apr 25;15:1335798. doi: 10.3389/fphys.2024.1335798 (PMC11084671; doi:10.3389/fphys.2024.1335798)
Supplement: Supplementary file 1 [file Table1.doc]

Supplementary Table 1. Postbronchodilator Spirometry results

| **Variable** | **Serious Mental Illness** | | |  | **Between Group Differences** | | |
| --- | --- | --- | --- | --- | --- | --- | --- |
|  |  |  |  |  | **(SMI total vs. Controls)** | | |
|  | **Total** | **Schizophrenia** | **Bipolar disorder** | **Controls** | **Difference** | **Cohen’s d** | **p value** |
|  |  |  |  |  | **(95% CI)** | **(95% CI)** |  |
| **PostBD FEV1, liters: mean (SD)** | 2.73 (0.75) | 2.83 (0.77) | 2.52 (0.69) | 3.04 (0.75) | 0.31 | 0.41 | 0.004 |
|  |  |  |  |  | (0.13, 0.49) | (0.18, 0.65) |  |
| **PostBD FEV1, % predicted: mean (SD)** | 85.9 (18.0) | 84.1 (18.9) | 89.6 (15.7) | 105 (16.8) | 19.10 | 1.09 | <0.001 |
|  |  |  |  |  | (14.96, 23.24) | (0.85, 1.33) |  |
| **PostBD FVC, liters: mean (SD)** | 3.62 (0.97) | 3.75 (0.99) | 3.37 (0.89) | 3.89 (0.94) | 0.27 | 0.28 | 0.048 |
|  |  |  |  |  | (0.04, 0.5) | (0.05, 0.52) |  |
| **PostBD FVC, % predicted: mean (SD)** | 89.3 (17.1) | 86.7 (16.3) | 94.5 (17.6) | 104 (15.5) | 14.7 | 0.89 | <0.001 |
|  |  |  |  |  | (10.81, 18.59) | (0.66, 1.13) |  |
|  | | | | | | | |

BD, bronchodilatation; FEV1, forced expiratory volume in 1 second; FVC, forced vital capacity; SD, standard deviation; SMI, serious mental illness
